# Regulation of hepatic insulin signaling and glucose homeostasis by sphingosine kinase 2

**DOI:** 10.1073/pnas.2007856117

**Published:** 2020-09-11

**Authors:** Gulibositan Aji, Yu Huang, Mei Li Ng, Wei Wang, Tian Lan, Min Li, Yufei Li, Qi Chen, Rui Li, Sishan Yan, Collin Tran, James G. Burchfield, Timothy A. Couttas, Jinbiao Chen, Long Hoa Chung, Da Liu, Carol Wadham, Philip J. Hogg, Xin Gao, Mathew A. Vadas, Jennifer R. Gamble, Anthony S. Don, Pu Xia, Yanfei Qi

**Affiliations:** ^a^Department of Endocrinology and Metabolism, Fudan Institute for Metabolic Diseases, Zhongshan Hospital, Fudan University, Shanghai 200032, China;; ^b^Centenary Institute, The University of Sydney, Sydney, NSW 2050, Australia;; ^c^Advanced Medical and Dental Institute, Universiti Sains Malaysia, Penang 13200, Malaysia;; ^d^School of Pharmacy, Guangdong Pharmaceutical University, Guangzhou 510006, China;; ^e^Department of Cardiology, Third Affiliated Hospital of Beijing University of Chinese Medicine, Beijing 100029, China;; ^f^School of Medical Sciences, The University of New South Wales, Sydney, NSW 2052, Australia;; ^g^School of Life and Environmental Sciences, The University of Sydney, Sydney, NSW 2006, Australia;; ^h^National Health and Medical Research Council Clinical Trials Centre, The University of Sydney, Sydney, NSW 2006, Australia;; ^i^National Clinical Research Center for Aging and Medicine, Huashan Hospital, Fudan University, Shanghai 200413, China

**Keywords:** hepatocyte, insulin resistance, sphingolipids, ceramide, type 2 diabetes

## Abstract

Sphingolipid dysregulation is often associated with insulin resistance, while the enzymes controlling sphingolipid metabolism are emerging as therapeutic targets for improving insulin sensitivity. We report herein that sphingosine kinase 2 (SphK2), a key enzyme in sphingolipid catabolism, plays a critical role in the regulation of hepatic insulin signaling and glucose homeostasis both in vitro and in vivo. Hepatocyte-specific *Sphk2* knockout mice exhibit pronounced insulin resistance and glucose intolerance. Likewise, SphK2-deficient hepatocytes are resistant to insulin-induced activation of the phosphoinositide 3-kinase (PI3K)-Akt-FoxO1 pathway and elevated hepatic glucose production. Mechanistically, SphK2 deficiency leads to the accumulation of sphingosine that, in turn, suppresses hepatic insulin signaling by inhibiting PI3K activation in hepatocytes. Either reexpressing functional SphK2 or pharmacologically inhibiting sphingosine production restores insulin sensitivity in SphK2-deficient hepatocytes. In conclusion, the current study provides both experimental findings and mechanistic data showing that SphK2 and sphingosine in the liver are critical regulators of insulin sensitivity and glucose homeostasis.

## Significance

Hepatic insulin resistance is a chief pathogenic determinant in the development of type 2 diabetes, which is often associated with abnormal hepatic lipid regulation. Sphingolipids are a class of essential lipids in the liver, where sphingosine kinase 2 (SphK2) is a key enzyme in their catabolic pathway. However, roles of SphK2 and its related sphingolipids in hepatic insulin resistance remain elusive. Here we generate liver-specific Sphk2 knockout mice, demonstrating that SphK2 in the liver is essential for insulin sensitivity and glucose homeostasis. We also identify sphingosine as a bona fide endogenous inhibitor of hepatic insulin signaling. These findings provide physiological insights into SphK2 and sphingosine, which could be therapeutic targets for the management of insulin resistance and diabetes.

The liver is a central organ in the regulation of whole-body glucose homeostasis under the fine-tuning by the anabolic hormone insulin (1). Upon nutrient uptake, insulin promotes glucose storage in the form of glycogen in the liver to avoid postprandial hyperglycemia; while in the fasted state, the liver supplies ∼90% of endogenous glucose via hepatic glucose production (HGP) when the insulin level is low (2, 3). However, hepatic insulin action is often impaired by aberrant lipid metabolites in obesity and many other pathological conditions (4). In accord, nonalcoholic fatty liver disease is present in 70−80% of type 2 diabetic subjects (5). Hepatic insulin resistance results in excess HGP, leading to hyperglycemia (2, 6). As such, to understand how lipids regulate hepatic insulin action is still a fundamental matter for the understanding of the pathogenesis of diabetes. By far, diacylglycerol and ceramide represent the most important candidates underpinning the mechanisms of lipid-induced insulin resistance (7). However, it is unlikely that diacylglycerol and ceramide are the only two lipid regulators of hepatic insulin signaling. The roles of many hepatic lipids and their metabolic enzymes in hepatic insulin resistance remain elusive.

Sphingolipids are a class of essential lipids, functioning as both cell membrane constituents and signaling messengers. Structurally, sphingolipids share a common backbone designated as a sphingoid base (8). In the sphingolipid metabolic network, ceramides serve as the central hub (8). Ceramides are biosynthesized from free fatty acids and reversibly converted to complex sphingolipids, such as sphingomyelin, glycosphingolipids, and acylceramides (8, 9). In the catabolic pathway, ceramides are hydrolyzed to sphingosine by acid, neutral, or alkaline ceramidase, followed by phosphorylation to sphingosine 1-phosphate (S1P) by sphingosine kinase (SphK), and eventually degraded into nonlipid products (9, 10). SphK is regarded as a “switch” of the sphingolipid rheostat, as it catalyzes the conversion of ceramide/sphingosine to S1P, which often exhibit opposing biological roles in the cell (11, 12). There are two human SphK isoforms, SphK1 and SphK2, which are encoded by two different genes. SphK2 is the dominant isoform in the liver (13). SphK1 and SphK2 have redundancy in some essential enzymatic activities, as the deletion of each gene has no fundamental defects in mice, whereas loss of both genes leads to embryonic lethality (14). However, SphK1 and SphK2 often exhibit different and even opposite functions in a context-dependent manner, perhaps due to their distinct tissue distribution, subcellular localization, and biochemical properties (10).

Unlike extensively studied SphK1, the pathophysiological roles of SphK2 are still poorly characterized. Only a few studies on the role of SphK2 in metabolic diseases have yet yielded inconsistent conclusions. We have recently reported that the global knockout of *Sphk2* (*Sphk2*^−/−^) ameliorates the diabetic phenotype by protecting pancreatic β-cells against lipoapoptosis (15). Besides, the deletion of *Sphk2* was recently shown to prevent aged mice from insulin resistance, at least in part, due to elevated adipose tissue lipolysis (16). On the other hand, Nagahashi et al. reported that *Sphk2*^−/−^ mice rapidly develop fatty livers after only a 2-wk high-fat diet (HFD) feeding (17). *Sphk2*^−/−^ also appears to predispose mice to alcoholic fatty liver disease (18). i.p. injection of FTY-720, a prodrug that is primed by SphK2 to function, improves hepatic steatosis and inflammation in high-cholesterol diet–induced nonalcoholic steatohepatitis model (19). Additionally, adenoviral overexpression of SphK2 in the liver improves glucose intolerance and insulin resistance in diet-induced obese mice (20). These studies indicate that SphK2 can function via either hepatic or extrahepatic approaches to influence whole-body metabolic homeostasis, leading to different outcomes in an experimental context-dependent manner. Therefore, the hepatocyte-autonomous role of endogenous SphK2 in insulin signaling and glucose homeostasis remains to be clarified.

In this study, we generated hepatocyte-specific *Sphk2* knockout (*Sphk2*-LKO) mice using the Cre-loxP strategy. *Sphk2*-LKO mice developed pronounced insulin resistance and glucose intolerance. In the absence of SphK2, hepatocytes were profoundly resistant to insulin-induced activation of phosphoinositide 3-kinase (PI3K)-Akt signaling and suppression of HGP. Mechanistically, we identified sphingosine as a bona fide inhibitor of hepatic insulin signaling. Blocking sphingosine production improved insulin resistance in SphK2-deficient hepatocytes, regardless of alterations in levels of ceramides and S1P. This study has demonstrated SphK2 as a player in the regulation of hepatic insulin sensitivity.

## Results

### Hepatocyte-Specific Knockout of *Sphk2* Alters Sphingolipid Metabolism in the Liver.

**Fig. 1.**
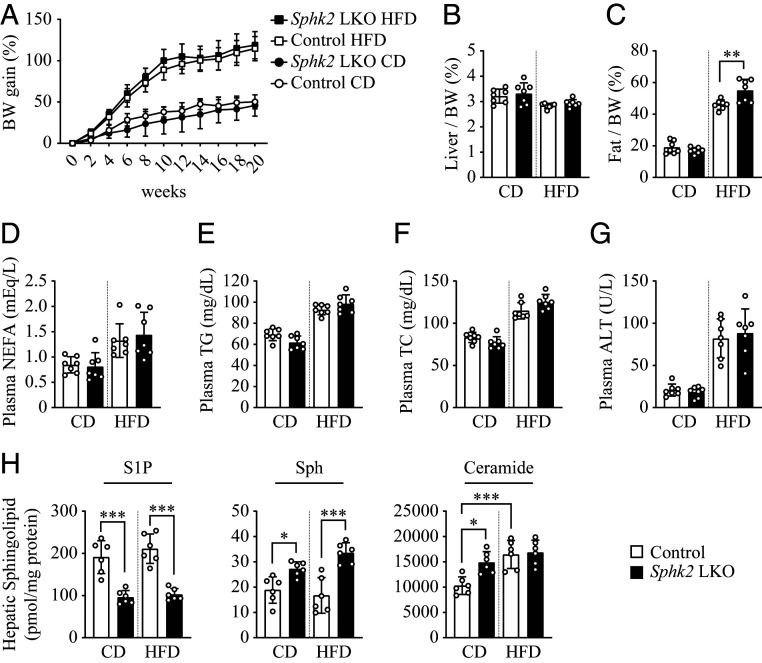
Physiological characteristics of *Sphk2*-LKO mice. (*A*) Body weight (BW) gain of hepatocyte-specific *Sphk2* knockout (*Sphk2*-LKO) and floxed control mice on a CD or HFD was monitored every other week for 20 wk. After 20 wk of feeding, mice were fasted for 16 h before sacrifice; *n* = 7. The liver weight (*B*) and fat weight (*C*) were normalized to body weight; *n* = 7. (*D*–*G*) Levels of NEFA (*D*), TG (*E*), TC (*F*), and ALT (*G*) in plasma; *n* = 7. (*H*) Levels of S1P, sphingosine (Sph), and ceramide mass in the liver; *n* = 6. Data are expressed as mean ± SD; *P < 0.05, **P < 0.01, ***P < 0.001.

To investigate the association of SphK2 and insulin resistance in the liver, we generated *Sphk2*-LKO mice. Loss of hepatic *Sphk2* displayed no difference in body weight gain (Fig. 1*A*) and the ratio of liver to body weight (Fig. 1*B*). Moreover, *Sphk2*-LKO did not affect plasma levels of nonesterified fatty acid (NEFA; Fig. 1*D*), triglyceride (TG; Fig. 1*E*), total cholesterol (TC; Fig. 1*F*), and alanine aminotransferase (ALT; Fig. 1*G*) on either chow diet (CD) or HFD. Upon the HFD feeding, *SphK2*-LKO mice exhibited a slightly increased adiposity (Fig. 1*C*). Due to the enzymatic function of SphK2 in converting ceramide/sphingosine to S1P, we determined the levels of these sphingolipids in livers (Fig. 1*H*). HFD feeding resulted in a significant increase in hepatic ceramide content, but it did not alter levels of S1P and sphingosine (Fig. 1*H*). Meanwhile, *SphK2*-LKO dramatically decreased S1P, but increased sphingosine content, under both feeding conditions (Fig. 1*H*). Interestingly, *SphK2*-LKO only increased the basal ceramide level, but not in diet-induced obese mice (Fig. 1*H*).

### Hepatocyte-Specific Knockout of *Sphk2* Impairs Insulin Sensitivity In Vivo.

**Fig. 2.**
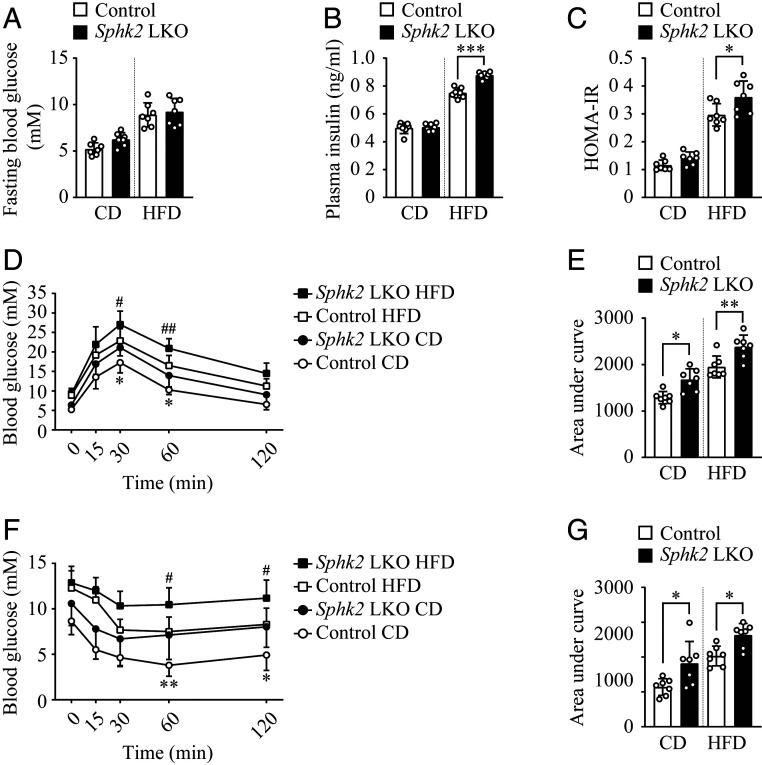
Hepatocyte-specific knockout of *Sphk2* results in glucose intolerance and insulin resistance. Hepatocyte-specific *Sphk2* knockout (*Sphk2*-LKO) and floxed control mice were fed on a CD or HFD for 20 wk. Levels of fasting blood glucose (*A*) and plasma insulin (*B*) were examined. (*C*) HOMA-IR score was calculated as fasting insulin (ng/mL) × fasting blood glucose (mM)/22.5. Oral glucose tolerance test (*D*) and i.p. insulin tolerance test (*F*) were performed and quantified as area under curve in (*E*) and (*G*), respectively. Data are expressed as mean ± SD; *P < 0.05, **P < 0.01, ***P < 0.001, *Sphk2*-LKO CD vs. Control CD, if not specified; ^#^*P* < 0.05, ^##^*P* < 0.01, *Sphk2*-LKO HFD vs. Control HFD; *n* = 7.

To assess if *Sphk2*-LKO affects insulin sensitivity and glucose homeostasis, we examined levels of fasting blood glucose (FBG) and plasma insulin and performed oral glucose tolerance test (GTT) and i.p. insulin tolerance test (ITT). *Sphk2*-LKO mice showed an increased trend in levels of FBG as compared to the control mice, whereas the data did not reach a statistical significance (Fig. 2*A*). However, *Sphk2*-LKO mice exhibited significantly elevated plasma insulin levels (Fig. 2*B*) and enhanced homeostasis assessment of insulin resistance (HOMA-IR) values under the HFD condition (Fig. 2*C*). Furthermore, *Sphk2*-LKO mice showed a reduced ability to dispose of i.p. glucose load from the circulation during oral GTT in both CD- and HFD-fed states (Fig. 2*D* and quantified as area under curve [AUC] in Fig. 2*E*). Correspondingly, *Sphk2*-LKO significantly reduced insulin sensitivity during ITT (Fig. 2*F* and quantified as AUC in Fig. 2*G*). These data all indicate that the deletion of *Sphk2* in hepatocytes led to pronounced glucose intolerance and insulin resistance, both typical prediabetic phenotypes.

### Knockout of *Sphk2* Impairs Insulin-Induced Suppression of Hepatic Gluconeogenesis.

**Fig. 3.**
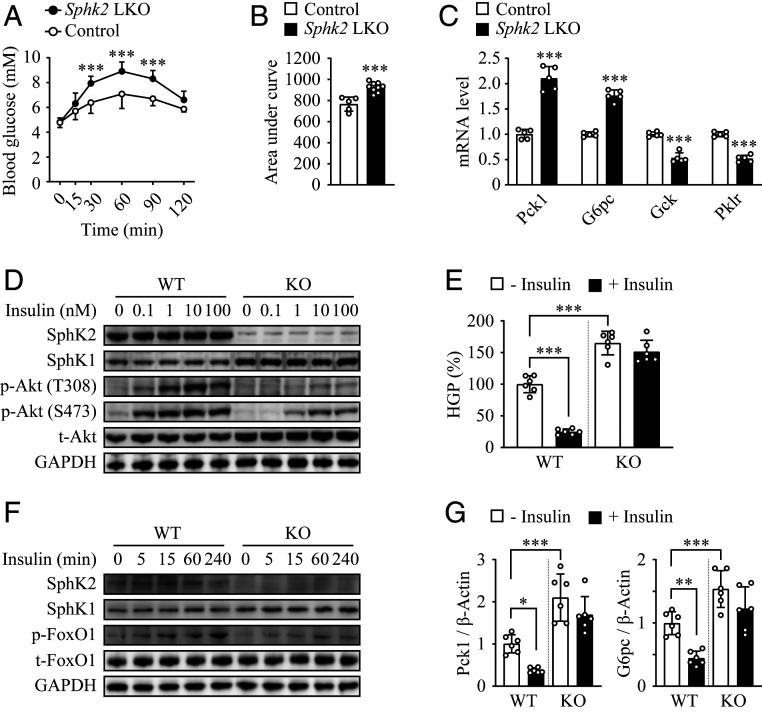
Knockout of *Sphk2* impairs insulin-mediated suppression of hepatic gluconeogenesis. (*A* and *B*) i.p. PTT was performed in hepatocyte-specific *Sphk2* knockout (*Sphk2*-LKO, *n* = 9) and floxed control (*n* = 5) mice on a normal chow diet (*A*), and quantified as area under curve (*B*). (*C*) mRNA expression of *Pck1, G6pc*, *Gck*, and *Pklr* were examined in liver tissues using RT-qPCR; *n* = 5. (*D*–*G*) Primary hepatocytes were isolated from WT and global *Sphk2* knockout (KO) mice. Western blot analyses were performed in cells stimulated with insulin at indicated concentrations for 15 min (*D*) or at 10 nM for indicated times (*F*). Primary hepatocytes were stimulated with 100 nM insulin for 6 h, then HGP (*E*) was examined in the culture medium, and mRNA expression levels of *Pck1* and *G6pc* (*G*) were quantified using RT-qPCR; *n* = 6. Data are expressed as mean ± SD; *P < 0.05, **P < 0.01, ***P < 0.001.

Suppression of gluconeogenesis through activation of the Akt/Fork Head Box O1 (FoxO1) signaling pathway is one of insulin’s primary actions in hepatocytes (2). We determined the hepatic glucose production in vivo by performing an i.p. pyruvate tolerance test (PTT). Administration of pyruvate, a gluconeogenic substrate, elevated the blood glucose level that was peaked at 60 min postinjection, by 23% and 85% in control and *Sphk2*-LKO mice, respectively (Fig. 3*A* and quantified as AUC in Fig. 3*B*). In addition, expression levels of gluconeogenic genes, phosphoenolpyruvate carboxykinase (*Pck1*) and glucose 6-phosphatase (*G6pc*), were up-regulated, whereas the genes involved in glucose utilization, glucokinase (*Gck*) and hepatic pyruvate kinase (*Pklr*), were down-regulated in *Sphk2*-LKO livers (Fig. 3*C*), which aligned with glucose intolerance and insulin resistance phenotype. To further define the role of SphK2 in hepatic gluconeogenesis, we examined insulin’s actions on gluconeogenesis and related signaling events in primary murine hepatocytes. In wild-type (WT) hepatocytes, insulin stimulation resulted in a significant increase in Akt phosphorylation at T308 and S473, hallmarks of insulin sensitivity, in a dose-dependent manner (Fig. 3*D*). In contrast, *Sphk2*^*−/−*^ hepatocytes responded to insulin to a much lesser extent (Fig. 3*D*). In accord, insulin suppressed glucose production by 75.3% in WT hepatocytes, whereas the insulin-induced suppression was abolished in *Sphk2*^*−/−*^ cells (Fig. 3*E*). It was associated with a similar change in FoxO1 phosphorylation (Fig. 3*F*) as well as messenger RNA (mRNA) levels of *Pck1* and *G6pc* (Fig. 3*G*). These data suggest a hepatocyte-autonomous role of SphK2 in insulin-mediated inhibition on gluconeogenesis.

### SphK2, but Not SphK1, Regulates Hepatic Insulin Signaling.

**Fig. 4.**
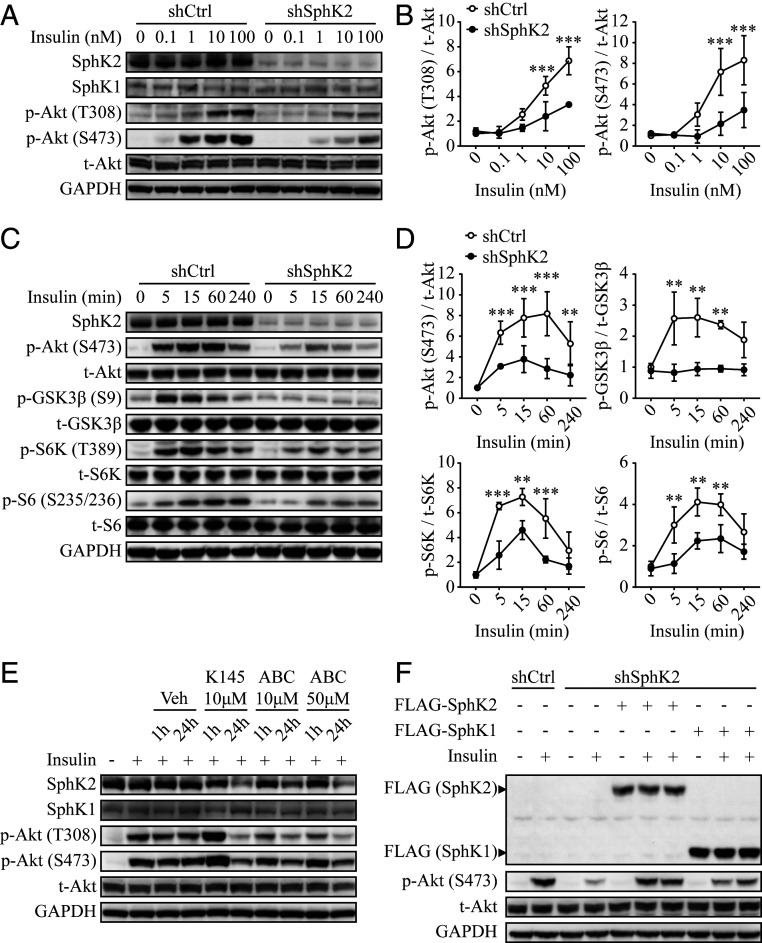
Knockdown of SphK2 impairs hepatic insulin signaling. SphK2 was knocked down in Huh7 hepatic cell line using lentiviral-based shRNA. Huh7 cells were treated with insulin at indicated concentrations for 15 min (*A*) or at 10 nM for indicated times (*C*). (*B* and *D*) Levels of indicated phosphorylated protein vs. total protein were quantified. Data are expressed as mean ± SD; **P < 0.01, ***P < 0.001, *n* = 3. (*E*) Huh7 parental cells were treated with vehicle (Veh; dimethyl sulfoxide), K145, or ABC294640 (ABC) for the indicated concentrations and times, prior to 15 min treatment with 10 nM insulin. (*F*) FLAG-tagged SphK1 or SphK2 were stably overexpressed in shSphK2 Huh7 cells. Cells were stimulated with 10 nM insulin for 15 min. Western blot analyses were performed.

Because SphK2 possesses some similar functional properties to SphK1, we sought to define whether the effect of SphK2 on hepatic insulin signaling is isoform-specific. To this end, we generated stable SphK1- and SphK2-knockdown Huh7 hepatic cell lines using lentiviral-based short-hairpin RNAs (shRNAs). In line with the above data obtained from murine *Sphk2*^*−/−*^ hepatocytes, knockdown of SphK2 significantly suppressed insulin-induced Akt phosphorylation on T308 and S473 in Huh7 hepatocytes (Fig. 4*A* and quantified in Fig. 4*B*) and also inhibited the phosphorylation of a panel of bona fide Akt effectors, including glycogen synthase kinase-3β (GSK3β), ribosomal protein S6 kinase, and S6, indicative of an impaired Akt activation pathway (Fig. 4*C* and quantified in Fig. 4*D*). In contrast, knockdown of SphK1 had little impact (*SI Appendix*, Fig. S1*A* and quantified in *SI Appendix*, Fig. S1*B*). The distinct effects of SphK1 and SphK2 on hepatic insulin signaling were also observed in another human hepatic cell line HepG2 (*SI Appendix*, Fig. S1*C*). Furthermore, we tested the effects of isoform-specific SphK inhibitors, PF-543, a highly selective inhibitor of SphK1 (21) as well as K145 and ABC294640, two selective inhibitors of SphK2 (22, 23). Consistent with gene knockdown results, we found that inhibition of SphK2 by K145 and ABC294640 dramatically decreased Akt phosphorylation after 24 h treatment (Fig. 4*E*), whereas inhibition of SphK1 by PF-543 exerted only a minimal effect (*SI Appendix*, Fig. S1*D*). Further, overexpression of SphK1 had marginal effects on insulin sensitivity in cells exposed to physiological doses of insulin and slightly increased phospho-Akt level upon treatment with insulin at a pharmacological concentration (*SI Appendix*, Fig. S1*E*). To rule out functional redundancy between SphK1 and SphK2, we reexpressed either SphK1 or SphK2 in the SphK2 knockdown Huh7 cells. Upon insulin stimulation, reexpression of SphK2 fully, whereas SphK1 scarcely, restored Akt phosphorylation in the SphK2-deficient hepatocytes (Fig. 4*F*). These data indicate that SphK2, but not SphK1, is primarily involved in the regulation of hepatic insulin signaling. Interestingly, knockdown of SphK2, but not SphK1, significantly increased sphingosine and decreased S1P levels (see Fig. 6*G* and *SI Appendix*, Fig. S1*F*). Furthermore, insulin was capable of stimulating the enzymatic activity of SphK2, but not SphK1, in hepatocytes (*SI Appendix*, Fig. S1*G*). These data suggest that distinct roles of SphK1 and SphK2 in the regulation of hepatic insulin signaling are likely hepatocyte-specific.

### Effect of SphK2 on Insulin-Induced PI3K Activation.

**Fig. 5.**
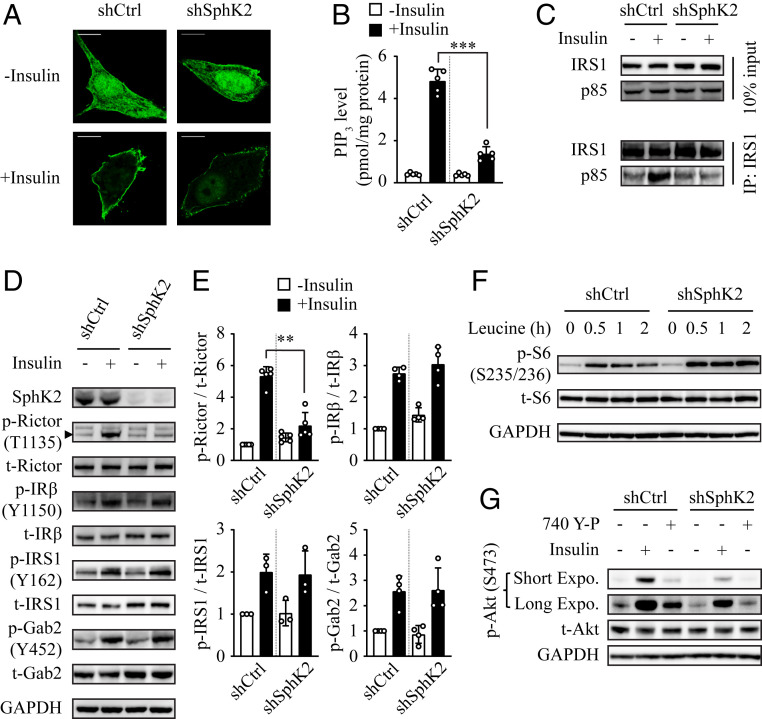
Effect of SphK2 on insulin-induced PI3K activation. SphK2 was knocked down in Huh7 cells using lentiviral-based shRNA. (*A*) PIP_3_ was visualized by the transfection of GFP-tagged Akt-PH. Bar, 10 μm. (*B*) PIP_3_ level was quantified using ELISA; *n* = 5. (*C*) Coimmunoprecipitation assay detecting the physical interaction between IRS1 and p85 subunit of PI3K. (*D* and *E*) Phosphorylation of the indicated proteins in insulin-signaling pathway was examined in cells treated with 10 nM insulin for 15 min by Western blot analyses (*D*) and normalized to total protein (*E*); *n* ≥ 3. (*F*) Level of phospho-S6 was examined in cells treated with 1 mM leucine for the indicated times. (*G*) Knockdown of SphK2 inhibited 740 Y-P–induced Akt phosphorylation. Cells were treated with 10 nM insulin or 25 μM 740 Y-P for 15 min. Data are expressed as mean ± SD; **P < 0.01, ***P < 0.001.

We next interrogated what the primary regulatory target of SphK2 in the hepatic insulin-signaling pathway was. Having demonstrated the effect of SphK2 on insulin-induced Akt phosphorylation, we examined a critical upstream signaling event, i.e., phosphatidylinositol 3,4,5-trisphosphate (PIP_3_) production by using an established fluorescent probe, GFP-Akt-PH (24). We found that insulin promoted PIP_3_ generation at the plasma membrane in control hepatocytes, but to a much lesser extent when SphK2 was knocked down (Fig. 5*A*). In accord, as quantified using the PIP_3_ ELISA, insulin increased PIP_3_ level by 11.7-fold in the control cells, which was significantly inhibited by SphK2 knockdown (Fig. 5*B*). We also examined PI3K activation by measuring the interaction of IRS1 and the p85 subunit of PI3K. A prominent physical interaction of IRS1 with p85-PI3K was detected upon insulin stimulation in control cells, whereas it was abrogated by SphK2 knockdown (Fig. 5*C*). In addition, the phosphorylation of rapamycin-insensitive companion of mammalian target of rapamycin (Rictor), downstream of PI3K activation, was significantly attenuated in SphK2 knockdown cells (Fig. 5*D* and quantified in Fig. 5*E*). Interestingly, the tyrosine phosphorylation of insulin receptor (IR), insulin receptor substrate 1 (IRS1), and growth factor receptor-bound protein 2 associated binding protein 2 (Gab2), upstream of PI3K activation, was unaltered in SphK2 knockdown cells (Fig. 5 *D* and *E*), indicating PI3K activation is the primary node regulated by SphK2 in hepatic insulin signaling. To further this notion, we treated cells with leucine that activates mammalian target of rapamycin complex 1 (mTORC1) and the downstream effector S6 in a PI3K/Akt-independent manner (25). While insulin-induced S6 phosphorylation was significantly inhibited (Fig. 4*C*), leucine was capable of stimulating S6 phosphorylation in SphK2 knockdown cells (Fig. 5*F*), indicating that the mTORC1 signaling downstream of PI3K/Akt remained intact. Furthermore, we treated cells with an activator of PI3K, the cell-permeable peptide 740 Y-P that mimics the effect of phosphorylated Tyr-IRS1. Treatment with 740 Y-P failed to activate Akt in SphK2 knockdown cells (Fig. 5*G*), further indicating the PI3K activation is the primary signaling node where SphK2 regulates hepatic insulin signaling.

### Sphingosine Is Primarily Responsible for the Effect of SphK2 on Hepatic Insulin Signaling.

**Fig. 6.**
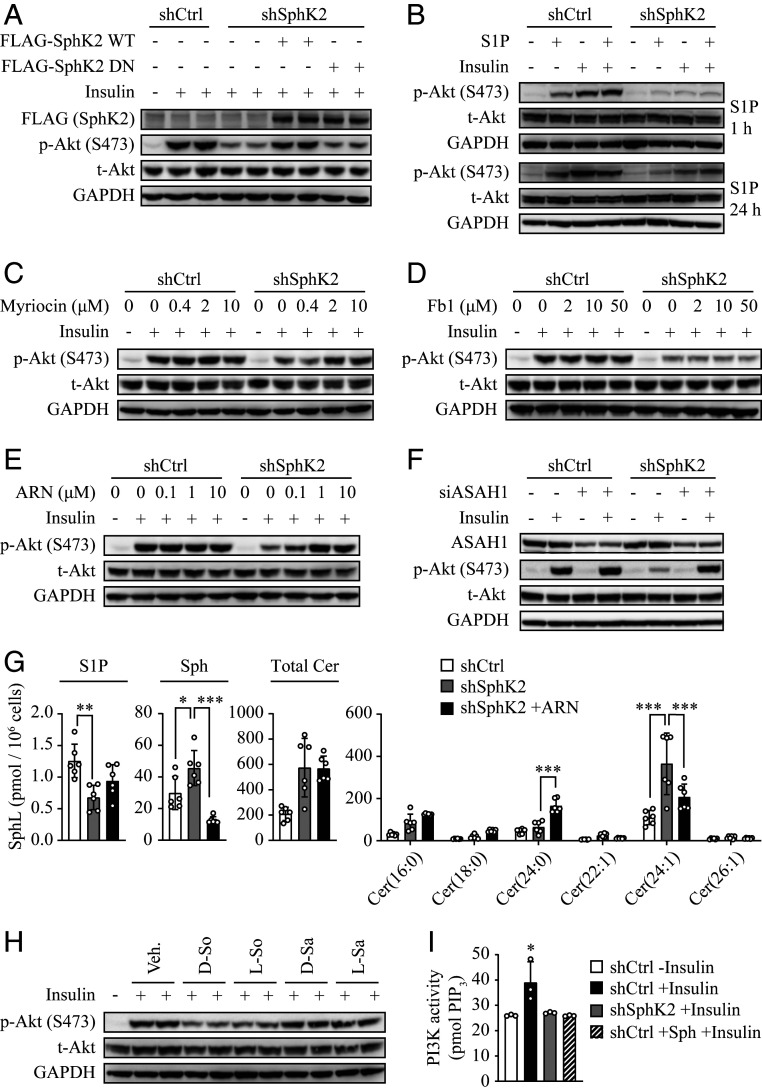
SphK2 regulates hepatic insulin signaling primarily via sphingosine. SphK2 was knocked down in Huh7 cells using lentiviral-based shRNA. (*A*–*F* and *H*) Western blot analyses were performed in cells treated with 10 nM insulin for 15 min following the indicated cotreatment. (*A*) FLAG-tagged WT-SphK2 or its DN mutant were stably overexpressed in shSphK2 Huh7 cells. (*B*–*E*) Cells were treated with 1 μM S1P for the indicated times (*B*), myriocin (*C*), fumonisin b1 (Fb1, *D*), or ARN14974 (ARN, *E*) at the indicated concentrations for 24 h. (*F*) Cells were transfected with negative control siRNA or siRNA against ASAH1 (siASAH1) for 48 h prior to insulin treatment. (*H*) Cells were treated with l- and D-form of sphingosine (So) or sphinganine (Sa) at 250 nM for 1 h. (*G*) Levels of ceramides (Cer), Sph, and S1P were quantified using lipidomics in indicated cells, untreated or treated with ARN14974 at 10 μM for 24 h; *n* = 6. (*I*) PI3K activity was examined following the treatment with 20 nM insulin for 15 min, in control cells, SphK2 knockdown (shSphK2) cells, and control cells pretreated with 250 nM sphingosine for 1 h; *n* = 3. Data are expressed as mean ± SD; *P < 0.05, **P < 0.01, ***P < 0.001.

SphK2 is a key enzyme in the sphingolipid catabolic pathway. We thus asked whether the enzymatic activity accounts for the effect of SphK2 on hepatic insulin signaling. In marked contrast to WT-SphK2, reexpression of a dominant-negative (DN) SphK2 (G248E) mutant was unable to restore the Akt phosphorylation in SphK2 knockdown cells, suggesting enzymatic activity of SphK2 played a critical role (Fig. 6*A*). We then sought to identify which sphingolipid species was primarily responsible for insulin resistance under SphK2 deficiency. We first examined if this resulted from a shortage of S1P. Surprisingly, treatment with S1P for either a short (1 h) or long (24 h) period had a negligible impact on Akt phosphorylation in SphK2 knockdown cells (Fig. 6*B*). We next treated cells with myriocin, a well-established inhibitor that blocks sphingolipid biosynthesis and thus reduces levels of all sphingolipid species, including S1P (26). Myriocin completely restored Akt activation in response to insulin (Fig. 6*C*), further ruling out the role of S1P. To segregate the impacts of ceramides and sphingosine, we treated cells with fumonisin b1 and ARN14974. Fumonisin b1 is a specific inhibitor of ceramide synthase, which reduces ceramides but increases sphingosine levels in cells (27), while ARN14974 is a novel inhibitor of acid ceramidase (ASAH1), which explicitly blocks ceramide conversion to sphingosine and thus reduces sphingosine content (28). Fumonisin b1 up to 50 μM failed to reverse Akt activation in SphK2 knockdown cells (Fig. 6*D*), whereas ARN14974 restored Akt phosphorylation in a dose-dependent fashion (Fig. 6*E*). In support of this, knockdown of ASAH1 by its specific small interfering RNA (siRNA) also rescued insulin sensitivity in SphK2-deficient cells (Fig. 6*F*). C16 ceramide has been suggested as a key pathogenic factor for hepatic insulin resistance in diet-induced obese mice (29, 30). Interestingly, ARN14974 substantially inhibited sphingosine production by 3.1-fold, whereas it did not significantly alter levels of C16 or total ceramide mass in SphK2 knockdown cells (Fig. 6*G*). Together, these data indicate that sphingosine, but not ceramides, is responsible for the inhibition of hepatic insulin signaling induced by SphK2 deficiency. To further this notion, we directly assessed the effect of sphingosine compared with its structural analog, sphinganine. While both l- and D-sphingosine profoundly suppressed insulin-induced Akt phosphorylation, neither l- nor D-sphinganine had such effects (Fig. 6*H*), supporting a specific role of sphingosine. Furthermore, sphingosine significantly inhibited the insulin-induced PI3K activation, to a similar extent as SphK2 knockdown in hepatocytes (Fig. 6*I*). Altogether, these data suggest that sphingosine is a bona fide endogenous inhibitor of hepatic insulin signaling and responsible for the effect of SphK2 in hepatocytes.

## Discussion

In the present study, we have uncovered a critical role of SphK2 in the regulation of hepatic insulin signaling. Hepatocyte-specific ablation of *Sphk2* impaired insulin metabolic action and glucose homeostasis, as evidenced by marked glucose intolerance, decreased insulin sensitivity, and hyperinsulinemia. Moreover, *Sphk2* knockout in hepatocytes disrupted the regulation of gluconeogenesis, as demonstrated by pyruvate intolerance in vivo and resistance to insulin-mediated suppression of HGP in vitro. Mechanistically, SphK2 deficiency suppressed hepatic insulin signaling by inhibiting PI3K activation. Furthermore, we identified sphingosine as a chief inhibitor of hepatic insulin signaling, which primarily accounts for the SphK2 deficiency-induced insulin resistance. Thus, this study has provided both functional and mechanistic evidence illustrating a player, SphK2, in the regulation of hepatic insulin signaling and metabolic action. We propose a working model for SphK2 function (*SI Appendix*, Fig. S2).

Studies on the in vivo role of SphK2 in liver metabolic function are limited, with discrepant results. SphK2 can be metabolically protective, as *Sphk2*^−/−^ predisposes to nonalcoholic and alcoholic fatty liver diseases, whereas adenoviral overexpression of SphK2 primarily in the liver improves insulin resistance (18–20). In contrast, SphK2 can be pathogenic, as systemic loss of *Sphk2* ameliorates diabetes by preventing pancreatic β-cell death and improves insulin sensitivity in aged mice by promoting lipolysis in adipose tissue (15, 16). These findings indicate that SphK2 exerts both hepatic and extrahepatic functions, leading to different metabolic outcomes in a context-dependent manner. Thus, it is critical to dissect SphK2’s functions in different tissues and their contribution to the overall metabolic homeostasis. To this end, we generated *Sphk2*-LKO mice, providing a powerful model to study the debated role of SphK2 in the liver. *Sphk2*-LKO mice developed profound insulin resistance and glucose intolerance (Fig. 2), which agrees with the previous report based on overexpression of SphK2 (20). It is worth noting that the effects of *Sphk2*-LKO were independent of the type of diet. Indeed, *Sphk2*-LKO on a regular diet exhibited an elevated hepatic glucose production upon pyruvate challenge (Fig. 3 *A* and *B*). Moreover, SphK2-deficient hepatocytes impaired insulin sensitivity in vitro, in the absence of a high-fat environment (Figs. 3–6). Interestingly, *Sphk2*-LKO resulted in increased adiposity and hyperinsulinemia under HFD feeding (Figs. 1*C* and 2*B*), suggesting *Sphk2*-LKO might cause extrahepatic impacts via interorganic communication. Under the hyperinsulinemia, FBG levels were not significantly elevated in *Sphk2*-LKO mice, indicating the mice remained in prediabetic stage during the experimental period. Both SphK1 and SphK2 are key enzymes in the sphingolipid catabolism pathway, converting ceramide and sphingosine into S1P (11). Of them, SphK2 is the dominant form of SphK in the liver (13). Our previous report has reported that the global knockout of *Sphk1* has minimal impact on insulin sensitivity (31). We found herein that SphK2, but not SphK1, was essential for hepatic insulin signaling (Fig. 4 and *SI Appendix*, Fig. S1), which supports the metabolically protective role of SphK2 in vivo, as shown in the *Sphk2*-LKO mice (Fig. 2).

Molecular components of the insulin-signaling pathway have been well established (32). We found that while insulin-induced PI3K/Akt activation was markedly blocked by SphK2 deficiency, there was no alteration in the tyrosine phosphoactivation machinery of IR and its adaptor proteins (Fig. 5). Consistent with this, overexpression of SphK2 elevates phospho-Akt level in primary murine hepatocytes, with no change in tyrosine phosphorylation of IRS1/2 (20). SphK2 is thus likely to act on a signal node downstream of IRS1/2 and upstream of Akt. Using various experimental strategies, we identified PI3K as a primary target for the action of SphK2 in the regulation of hepatic insulin signaling. SphK2 deficiency resulted in 1) disrupted PI3K-IRS1 interaction, 2) decreased PI3K activity, 3) reduced PIP_3_ generation, and 4) inhibition of PI3K activator-mediated signaling (Figs. 5 *A*–*C* and *G* and 6*I*). However, to the best of our experimental skills, we have not convincingly detected direct interactions of SphK2 with the molecular components of PI3K or the related phosphatidylinositol metabolites, which remains to be addressed in the future.

Sphingolipids have been widely implicated in the pathogenesis of diabetes and insulin resistance (33–35). However, compared to other tissues, the liver appears to possess a distinct subset of sphingolipid metabolites associated with insulin resistance (36, 37). Whether and which sphingolipids regulate hepatic insulin sensitivity remain enigmatic. The findings that inhibition of SphK2 by pharmaceutical inhibitors or genetic means (DN mutation) profoundly blocked insulin signaling and action in hepatocytes, which indicates that the catalytic properties of SphK2 are crucial (Figs. 4*E* and 6*A*). SphK2 often functions in the cell via its catalytic product S1P (38, 39), and S1P is capable of inducing Akt phosphorylation via various signaling pathways (20, 40). In line with this, S1P induced Akt phosphorylation in hepatocytes (Fig. 6*B*). However, S1P was inadequate to restore insulin response in SphK2 knockdown hepatocytes (Fig. 6*B*). Surprisingly, myriocin that reduces the S1P level could fully restore insulin sensitivity in SphK2-deficient cells (Fig. 6*C*), strongly indicating that S1P is irrelevant to this regulation. Because myriocin is a specific inhibitor of serine palmitoyltransferase that is responsible for catalyzing the committed step of sphingolipid biosynthesis, its ability to restore the inhibition of insulin signaling suggests an accumulation of particular sphingolipid species that may serve as negative regulators. Ceramides, particularly C16 ceramide, have been commonly recognized as a negative regulator of insulin signaling (29, 30). However, the role of ceramides in the liver is controversial, as its hepatic levels are sometimes unrelated to hepatic insulin sensitivity in humans and rodents (reviewed in ref. 7). Indeed, hepatic ceramide levels were comparable in control and *Sphk2*-LKO mice on HFD, but *Sphk2*-LKO mice exhibited more severe insulin resistance (Fig. 1*H*). Also, fumonisin b1 that inhibits ceramide production failed to improve insulin resistance in SphK2 knockdown cells (Fig. 6*D*). These results suggest that ceramides are unlikely to be responsible for insulin resistance in SphK2-deficient hepatocytes.

Sphingosine, the central substrate of SphK2 in hepatocytes, is mainly produced via ASAH1-mediated hydrolysis of ceramides (41). By blocking this pathway, the ceramidase inhibitor ARN14974 is known to elevate the ceramide level and decrease the sphingosine level in the cell (28). Interestingly, both ARN14974 and siRNA-mediated knockdown of ASAH1 significantly rescued insulin sensitivity in SphK2-deficient hepatocytes (Fig. 6 *E* and *F*). Meanwhile, ARN14974 dramatically reduced levels of sphingosine, but not C16 or total ceramide, in SphK2-deficient cells, indicating that the accumulation of sphingosine may chiefly account for the effect of SphK2 deficiency (Fig. 6*G*). The inhibitory effect of sphingosine on hepatic insulin signaling was further confirmed by the experiments using various sphingosine compounds, which shows that sphingosine, but not sphinganine, has a potent effect, inhibiting insulin-induced Akt phosphorylation and PI3K activity in hepatocytes (Fig. 6 *H* and *I*). However, how sphingosine inhibits PI3K remains unknown. It has been demonstrated that sphingosine can both physically and functionally interact with the protein 14-3-3ζ (42), which, in turn, regulates plasma membrane recruitment and activation of PI3K (43, 44). To what extent this pathway contributes to the regulation of hepatic insulin signaling is worthy of further investigation.

In summary, the current study has provided both experimental and mechanistic data implicating a critical role of SphK2 in hepatic insulin signaling. Specifically, the ablation of *Sphk2* in hepatocytes led to insulin resistance both in vivo and in vitro. Interestingly, a decreased hepatic level of SphK2 expression was found in human type 2 diabetic subjects (Gene Expression Omnibus profile ID#71277852). In addition, we identified sphingosine as a bona fide endogenous inhibitor of hepatic insulin signaling. Restoration of SphK2 expression and pharmacological depletion of sphingosine levels substantially improved hepatic insulin sensitivity, which provides a potential therapeutic option against diabetes.

## Materials and Methods

### Animals.

All mice are on a C57BL/6 background. The *Sphk2-*LKO mice were generated by cross-breeding *Albumin-Cre*^*Tg*/+^ mice (Jackson Laboratories) with mice homozygous for a “floxed” exon 2 of *Sphk2* (*Sphk2*^*fl//fl*^) by Cyagen. All experiments involving *Sphk2-*LKO mice were approved by the Animal Use and Care Committees of Fudan University and Guangdong Pharmaceutical University, China, and confirmed with the US Public Health Service Policy on Humane Care and Use of Laboratory Animals. *AlbCre* progressively excises the floxed gene in mouse hepatocytes until a complete deletion at 6 wk of age (45). Thus, male floxed *Sphk2* and *Sphk2*-LKO mice aged 6–8 wk were randomly assigned to be fed with either a CD or HFD (containing 60 kcal% fat, 20% protein, and 20% carbohydrate; Research Diets) for 20 wk. Mice were maintained in a 12-h light/dark cycle, allowed food and water ad libitum. Levels of plasma insulin (Insulin ELISA kit, Millipore), NEFA, TG, TC (WAKO kits), and ALT (ELISA Kit, TW-REAGENG) were measured after 16 h starvation. The use of global *Sphk2*^*−/−*^ mice, gifts from Richard Proia, The National Institute of Diabetes and Digestive and Kidney Diseases, National Institutes of Health (NIH) (14), was approved by Research Ethics and Governance Office, Royal Prince Alfred Hospital, Australia.

### Cell Culture.

Huh7 hepatic cell lines were obtained from CellBank Australia, while HepG2 hepatic cell line and 3T3-L1 preadipocytes were obtained from American Type Culture Collection. Primary hepatocytes were isolated from male mice aged 10–12 wk, using collagenase perfusion and subsequent Percoll gradient centrifugation (46). Cells were all maintained in Dulbecco's modified Eagle medium (DMEM) supplemented with 10% fetal calf serum and 100 units/mL penicillin/streptomycin. To induce differentiation to adipocytes, we cultured 3T3-L1 preadipocytes in DMEM containing 10% fetal bovine serum, 1% penicillin/streptomycin, 5 μg/mL insulin, 1 μM dexamethasone, and 0.5 mM isobutylmethylxanthine (47). The fetal calf serum was deprived overnight prior to the treatment with insulin.

### Statistics.

Comparisons between two groups were analyzed by unpaired two-tailed *t* tests, and multiple comparisons were analyzed by ANOVA with Tukey tests, using GraphPad Prism 8.4. Differences at values of *P* < 0.05 were considered significant.

## Supplementary Material

Supplementary File

## Data Availability

The authors declare that there are no restrictions on data or material availability. All data supporting the findings of this study are contained in the manuscript text and *SI Appendix*.
