# Supplementary material for: Regulation of hepatic insulin signaling and glucose homeostasis by sphingosine kinase 2
Source: Proc Natl Acad Sci U S A. 2020 Sep 11;117(39):24434–42. doi: 10.1073/pnas.2007856117 (PMC7533871; doi:10.1073/pnas.2007856117)
Supplement: Supplementary File [file pnas.2007856117.sapp.pdf]

Supplemental Figure 1

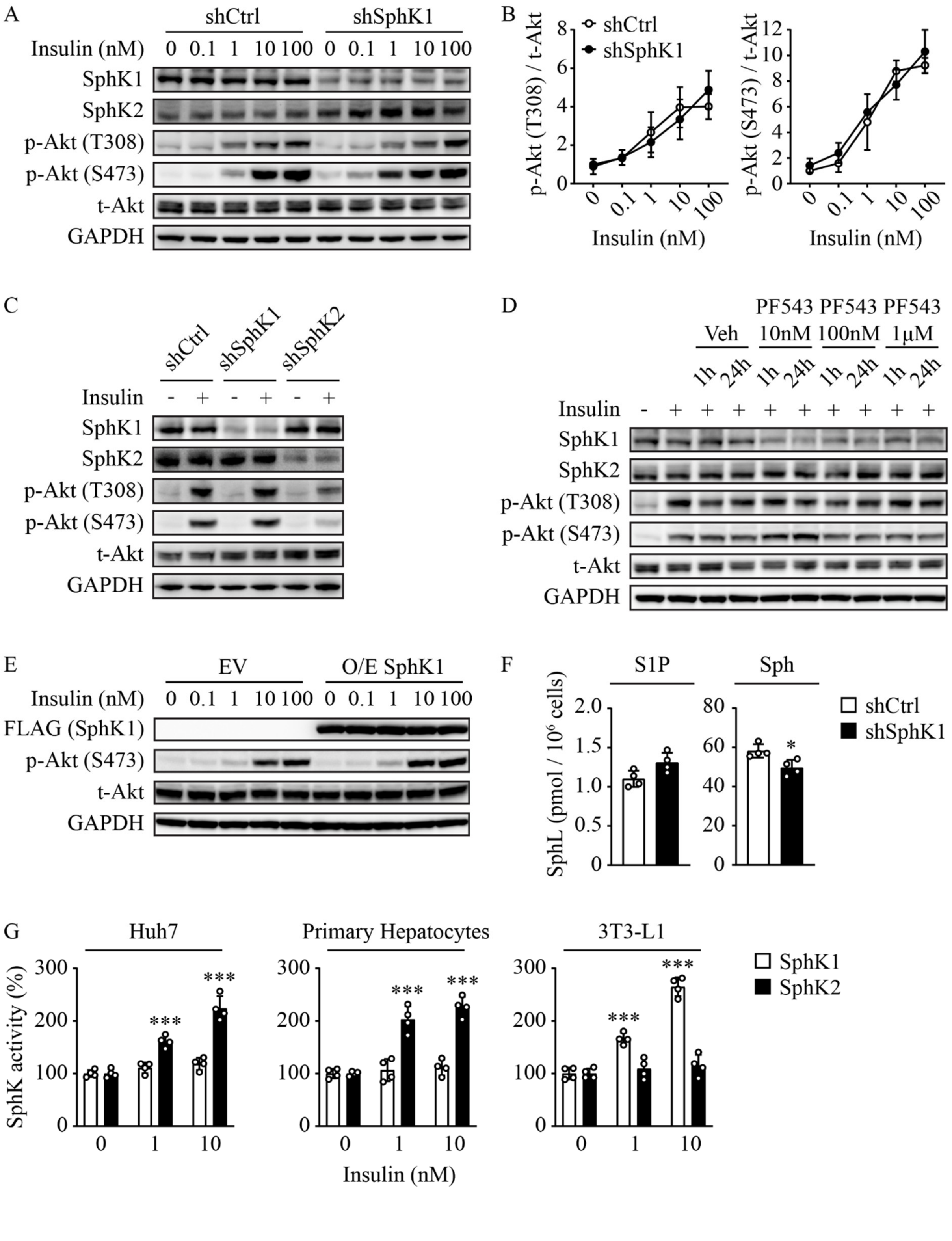

Supplemental Figure 1 SphK1 has little impact on hepatic insulin signaling. SphK1 or SphK2 was knocked down in hepatic cell lines using lentiviral-based short hairpin RNA (shRNA).

A,B Huh7 cells were treated with insulin at the indicated concentrations for 15 min. Western blot analyses were performed (**A**), and the level of Akt phosphorylation (p-Akt) versus total Akt (t-Akt) expression was quantified (**B**); n = 3. **C** HepG2 cells were treated with 10 nM insulin for 15 min. **D** Huh7 parental cells were treated with vehicle (Veh; DMSO) or PF543 for the indicated concentrations and times, prior to 15 min treatment with 10 nM insulin. **E** Huh7 parental cells were transfected with empty vector (EV) or the plasmid encoding FLAG-tagged SphK1 for 48 h prior to the treatment with insulin at indicated concentrations for 15 min. **F** Levels of S1P and sphingosine (Sph) were examined by targeted lipidomics; n = 4. **G** SphK activity was examined in Huh7 cells, primary murine hepatocytes and 3T3-L1 adipocytes upon insulin stimulation at indicated concentrations; n = 4. Data are expressed as means ± SD. * $p<0.05$, *** $p<0.001$, vs. untreated control.

Supplemental Figure 2

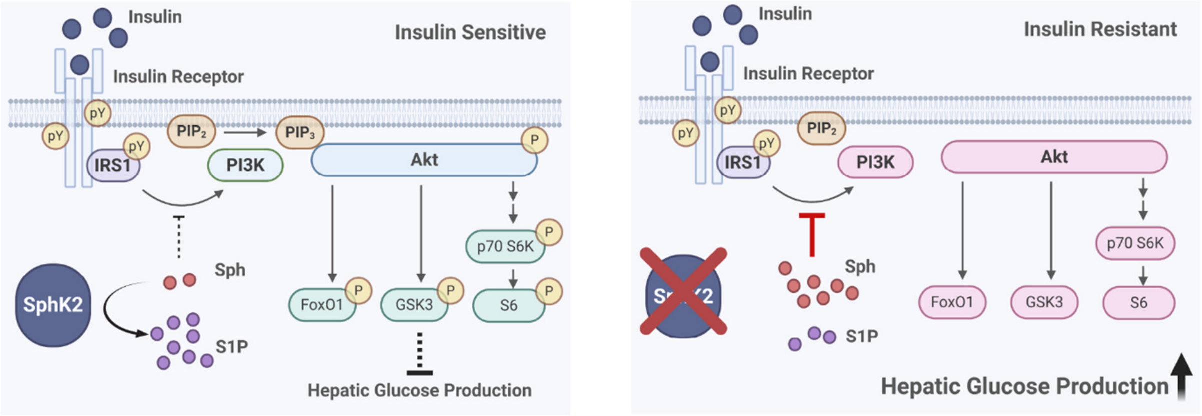

Supplemental Figure 2 Model depicting the role of SphK2 in the regulation of hepatic insulin signaling. **Left panel** Under basal conditions (e.g., WT mice), SphK2 regulates the homeostasis of sphingosine in hepatocytes, which is required for maintaining normal insulin signaling and actions in the liver. **Right panel** Under certain pathological conditions (e.g., *Sphk2*-LKO mice), the accumulation of sphingosine suppresses PI3K activation and Akt signaling, which results in insulin resistance and glucose intolerance.

Supplemental Materials and Methods

Glucose tolerance test (GTT), Insulin tolerance test (ITT) and pyruvate tolerance test (PTT). Following overnight fasting, mice were administered by gavage with D-glucose (Sigma) at 2 g/kg body weight for GTT or intraperitoneally injected with pyruvate (Sigma) at 2 g/kg body weight for PTT. In ITT, mice were fasted for 6 h prior to intraperitoneal injection with insulin (Sigma) at 0.5 IU/kg body weight. Glucose levels were measured from tail bleeds using a glucometer at specified time points during tests, during the light cycle.

Glucose production assay. Primary murine hepatocytes were treated with 100 nM insulin for 6 h in glucose-free medium (Sigma #D5030) supplemented with 20 mM sodium lactate, 2 mM sodium pyruvate, 2 mM L-glutamine and 15 mM HEPES (1). The glucose production in the culture medium was determined using the Amplex® Red Glucose/Glucose Oxidase Assay Kit (Thermo Fisher Scientific).

Real-time quantitative PCR. RNA extraction and reverse transcription were performed, as previously described (2). Total RNA was extracted using TRIZol reagent (Thermo Fisher Scientific) and chloroform (Sigma), precipitated in 2-propanol (Sigma), washed in ethanol (Sigma), and quantified using NanoDrop (Thermo Fisher Scientific). mRNA was reverse transcribed into cDNA using High-Capacity cDNA Reverse Transcription Kit (Thermo Fisher Scientific), according to the manufacturer protocol. mRNA level was quantified on a Roche Lightcycler 480 PCR machine using KAPA SYBR® FAST (Sigma). Primer sets used for PCR are mouse *Pck1* F-gctggccccgggagtcacc, R-tgccgaagttgtagccgaagaagg (3); mouse *G6pc* F-tctttcccatctggttccatc, R-gtttggacaacgcccgtatt (3); mouse *Gck* F-gagatggatgtggtggcaat, R-accagctccacattctgcat (4); mouse *Pklr* F-ttcttccagcagcagcaac, R-tcatctccttgaggcggtc (5); mouse β-actin F- ccttcttgggtatggaatcctg, R-cagtaatctccttctgcatcctg.

shRNA, Plasmids and siRNA

shRNAs targeting human SphK1 (TRCN0000333675) and SphK2 (TRCN0000036973 and TRCN0000359274) were constructed in pLKO.1-puro Lentiviral vector (Sigma). The lentivirus was produced in HEK293T cells using plasmids gifted from Dr Didier Trono through Addgene, including pMD2.G, pMDLg/pRRE and pRSV-Rev (6). Lentiviral transduction was carried out, as described previously (7). In brief, shRNA and packaging plasmids were transfected in HEK293T cells. The lentivirus-containing medium was collected at 48 h after the plasmid transfection, filtered through 0.45 μm filter (Millipore), supplemented with 8 μg/ml polybrene (Sigma) and introduced to parent cell culture. The shSphK2 (TRCN0000359274) was used for WT and mutant SphK2 re-expression, as it targets 3'UTR. Flag-tagged human SphK1 (NM_001142601) and SphK2 (NM_020126) plasmids in pcDNA3.1/Zeo(+) vector were obtained from GenScript. GFP-Akt-PH for probing PIP_3 was a gift from Dr Tamas Balla through Addgene (8). Plasmid transfection was carried out using Lipofectamine LTX plus reagents (Thermo Fisher Scientific), following the manufacturer's protocol. siRNA-mediated ASAH1 knockdown was performed using Lipofectamine RNAiMax reagent (Thermo Fisher Scientific) complexed with predesigned siRNA against ASAH1 (Sigma).

Treatment. Human insulin solution and leucine were purchased from Sigma. D-erythro-S1P and D-erythro-sphinganine from Avanti Polar Lipids. PF543, K145, myriocin, fumonisin B1, ARN14974, 740 Y-P, L-erythro-sphingosine, and L-erythro-sphinganine from Cayman Chemical. D-erythro-sphingosine and ABC294640 were supplied by Echelon Biosciences.

Immunoblotting and co-immunoprecipitation. Proteins were extracted with cell lysis buffer containing 50 mM HEPES pH 7.4, 150 mM NaCl, 10% glycerol, 1% Triton X-100,

PHOSSTOP (Sigma) and cOmplete™ ULTRA Protease Inhibitor Cocktail (Sigma), sonicated using QSonica-Q800R2, quantified using bicinchoninic acid assay (Sigma) and separated using NuPAGE™ 4-12% Bis-Tris precast gels (Thermo Fisher Scientific). Immunoblotting was conducted with the following antisera: SphK1, SphK2 (human-specific), p-Akt-S473, p-Akt-T308, t-Akt, GAPDH, p-FoxO1, t-FoxO1, t-IRS1, p85, p-RICTOR-T1135, t-RICTOR, p-IRβ-Y1150/1151, t-IRβ, p-Gab2-Y452, t-Gab2, p-GSK3β-S9, t-GSK3β, p-P70S6K-T389, t-P70S6K, p-S6-S235/236, t-S6 from Cell Signaling Technology; SphK2 (reactive in mouse) and ASAH1 from Proteintech; β-actin and FLAG from Sigma; p-IRS1-Y612 from R&D Systems. To immunoprecipitate IRS1, the t-IRS1 (polyclonal antibody from Cell Signaling Technology) was immobilized on Protein A Sepharose beads (GE Healthcare). The immunoprecipitates were eluted in LDS sample buffer (Thermo Fisher Scientific). Chemiluminescence was detected with a BIORAD ChemiDoc TOUCH imaging system.

SphK activity assay. SphK activity was determined using 20 μM N-(7-nitro-2–1,3-benzoxadiazol-4-yl)-d-erythro (NBD)-sphingosine (Cayman Chemical) as substrates and 1 mM ATP (Sigma), as described previously (9). After incubation for 30 min, NBD-S1P was extracted with chloroform : methanol (2:1) at pH 8.5, and the fluorescence was determined with excitation at 485 nm and emission at 538 nm. To discriminate SphK1 and SphK2 activity, we added potassium chloride (1 M) and Triton X-100 (0.25%) to inhibit SphK1 or SphK2 activity, respectively.

Confocal microscopy. The confocal microscopy was performed using a Nikon C2 microscope. PIP_3 was probed by the transfection of GFP-Akt-PH. The images were processed using FIJI Image J software.

PIP_3 ELISA and PI3K activity assay. PIP_3 was quantified using PIP_3 Mass ELISA kit (Echelon), following the manufacturer's protocol with minor modifications. To increase the efficiency of lipid extraction, we sonicated samples for 20 min with the intermittent vortex. PI3K activity was determined using PI3-Kinase Activity ELISA Pico kit (Echelon), following immunoprecipitation of PI3K using p85 antibody (Cell Signaling Technology) and incubation with phosphatidylinositol 4,5-bisphosphate (PIP_2) substrates for 4 h at 37 °C.

Measurement of sphingolipids. Ceramides, sphingosine and S1P were analyzed, as described previously (10). In brief, sphingosine and S1P were quantified on a Thermo Fisher TSQ Altis triple quadrupole mass spectrometer, operated in positive ion mode, coupled to a Vanquish UHPLC system (Thermo). Lipids were separated on an Agilent Eclipse Plus C8 column. Peaks were integrated using Xcalibur (Thermo Fischer). Ceramides in Huh7 cells were quantified using untargeted lipidomic profiling on a Q Exactive HF-X mass spectrometer, with heated electrospray ionization (HESI), coupled to a Vanquish UHPLC system (ThermoFisher). Ceramides were resolved on a Waters Acquity C18 UPLC column. LipidSearch software v4.1.30 (Thermo Fisher) was used for lipid annotation, chromatogram alignment, and peak integration from extracted ion chromatograms. Ceramide mass in liver tissues was examined using targeted lipidomics.

SI References

1. M. Sakai *et al.*, CITED2 links hormonal signaling to PGC-1alpha acetylation in the regulation of gluconeogenesis. *Nat Med* **18**, 612-617 (2012).
2. Y. Qi *et al.*, CDP-diacylglycerol synthases regulate the growth of lipid droplets and adipocyte development. *J Lipid Res* **57**, 767-780 (2016).
3. N. J. Poritsanos, P. S. Lew, T. M. Mizuno, Relationship between blood glucose levels and hepatic Fto mRNA expression in mice. *Biochem Biophys Res Commun* **400**, 713-717 (2010).
4. E. M. Allister *et al.*, UCP2 regulates the glucagon response to fasting and starvation. *Diabetes* **62**, 1623-1633 (2013).
5. T. Suhara *et al.*, Inhibition of the oxygen sensor PHD2 in the liver improves survival in lactic acidosis by activating the Cori cycle. *Proc Natl Acad Sci U S A* **112**, 11642-11647 (2015).
6. T. Dull *et al.*, A third-generation lentivirus vector with a conditional packaging system. *J Virol* **72**, 8463-8471 (1998).
7. M. Pagac *et al.*, SEIPIN Regulates Lipid Droplet Expansion and Adipocyte Development by Modulating the Activity of Glycerol-3-phosphate Acyltransferase. *Cell Rep* **17**, 1546-1559 (2016).
8. P. Varnai, T. Balla, Visualization of phosphoinositides that bind pleckstrin homology domains: calcium- and agonist-induced dynamic changes and relationship to myo-[3H]inositol-labeled phosphoinositide pools. *J Cell Biol* **143**, 501-510 (1998).
9. A. Billich, P. Ettmayer, Fluorescence-based assay of sphingosine kinases. *Anal Biochem* **326**, 114-119 (2004).
10. N. Turner *et al.*, A selective inhibitor of ceramide synthase 1 reveals a novel role in fat metabolism. *Nat Commun* **9**, 3165 (2018).
